# Identifying the Links Between Trauma and Social Adjustment: Implications for More Effective Psychotherapy With Traumatized Youth

**DOI:** 10.3389/fpsyg.2021.666807

**Published:** 2021-04-20

**Authors:** Sayedhabibollah Ahmadi Forooshani, Kate Murray, Nigar Khawaja, Zahra Izadikhah

**Affiliations:** ^1^School of Psychology and Counselling, Queensland University of Technology, Brisbane, QLD, Australia; ^2^School of Psychology and Counselling, University of Southern Queensland, Ipswich, QLD, Australia

**Keywords:** trauma, thought suppression, autobiographical memory, social problem solving, social adjustment

## Abstract

**Background:** Past research has highlighted the role of trauma in social adjustment problems, but little is known about the underlying process. This is a barrier to developing effective interventions for social adjustment of traumatized individuals. The present study addressed this research gap through a cognitive model.

**Methods:** A total of 604 young adults (aged 18–24; living in Australia) from different backgrounds (refugee, non-refugee immigrant, and Australian) were assessed through self-report questionnaires. The data were analyzed through path analysis and multivariate analysis of variance. Two path analyses were conducted separately for migrant (including non-refugee and refugee immigrants) and Australian groups.

**Results:** Analyses indicated that cognitive avoidance and social problem solving can significantly mediate the relation between trauma and social adjustment (*p* < 0.05). The model explaining this process statistically fit the data (e.g., NFI, TLI, CFI > 0.95). According to the model, reacting to trauma by cognitive avoidance (i.e., chronic thought suppression and over-general autobiographical memory) can disturb the cognitive capacities that are required for social problem solving. Consequently, a lack of effective social problem solving significantly hinders social adjustment. There were no significant differences among the Australian, non-refugee immigrant and refugee participants on the dependent variables. Moreover, the hypothesized links between the variables was confirmed similarly for both migrant (including refugee and non-refugee immigrants) and Australian groups.

**Conclusion:** The findings have important implications for interventions targeting the social adjustment of young individuals. We assert that overlooking the processes identified in this study, can hinder the improvement of social adjustment in young adults with a history of trauma. Recommendations for future research and practice are discussed.

## Introduction

Experiencing trauma during childhood and adolescence can lead to subsequent impairments in social adjustment (Higgins and McCabe, 2000; Tyler, 2002; Patel et al., 2017). Although posttraumatic stress symptoms can be considerably improved by psychological interventions (Brown et al., 2017), difficulties with social adjustment may remain unimproved in traumatized youths (Ahmadi Forooshani et al., 2019). When social adjustment problems are not improved, it can result in major risks for mental health problems such as increased vulnerability to depression and anxiety (Bosc et al., 1997; Montgomery and Foldspang, 2008). The issues related to social adjustment can especially be expected amongst youth populations (Rousseau et al., 2004), because adolescence and young adulthood represent critical stages of development in terms of the rapid changes, and adjusting with new social roles, norms, and expectations (Berk, 2007). Therefore, it is important to understand why difficulties with social adjustment in traumatized youth are resistant to improve. It seems that there is a complex relation between trauma and social adjustment that has been overlooked in trauma-focused interventions (McLean et al., 2013; Ahmadi Forooshani et al., 2019). In this study, we proposed and investigated a cognitive model to explain how trauma may result in difficulties with social adjustment.

According to our hypothesized model, experiencing trauma can disturb key cognitive capacities that are necessary for social adjustment. As such, when these capacities remain unimproved, significant issues with social adjustment may continue to persist. We argue that overlooking this process is the reason why psychological interventions have not been successful in addressing social adjustment of traumatized youth (Ahmadi Forooshani et al., 2019). The details of the theoretical framework of our hypothesized model are presented in the next section.

### The Theoretical Framework

We reviewed studies from multiple theoretical and research disciplines to identify and conceptualize a specific cognitive process between trauma and social adjustment. In this section, it is explained step by step how certain reactions to trauma can sequentially impair capacities of social adjustment.

A common reaction to trauma is avoiding trauma-related thoughts and memories (American Psychiatric Association, 2013). This can protect the person from unpleasant intrusive thoughts and memories as well as their associated negative emotions, but these strategies are only beneficial in the short-term (Wegner and Zanakos, 1994; Williams, 1996). When this type of cognitive avoidance in thoughts and memories is used chronically, it can change into dominant maladaptive cognitive functions (Sutherland and Bryant, 2007; Vazquez et al., 2008). One of these functions is chronic thought suppression which means a pervasive tendency to avoid processing negative thoughts (Najmi, 2013). Chronic thought suppression is not limited to traumatic events and it can be generalized to all subjects of personal or interpersonal life so that the person will not be able to process many negative or challenging aspects of their life (Wegner and Zanakos, 1994).

Similarly, when the person continuously avoids recalling traumatic memories, an impairment can be observed in recalling non-traumatic memories as well. In this case, individuals with trauma history tend to recall their past memories in a general way and without specific details (Sutherland and Bryant, 2007). This is considered an automatic and passive protective reaction against unpleasant emotions and intrusive thoughts that are associated with traumatic memories. This is because when access to past memories (including negative, positive, or neutral memories) is not specific and clear, the details of traumatic memories cannot be recalled specifically either (Williams, 1996; Williams et al., 2007). The memory function that is impaired through this process is called autobiographical memory. It refers to the ability to remember personal experiences and events of one's own life either generally or with detail (Raes et al., 2009).

All autobiographical memories can be divided into two main categories: Specific or over-general. Specific memories are memories that the person remembers with details including place and time. The duration of these memories is not more than a day. These memories can be either personally important or not; recent or related to a long time ago (Williams et al., 2007). In contrast, non-specific recalling of autobiographical memories is called over-general memory. Over-general memories are general and vague memories that cover an extended period of time (i.e., more than 1 day) or a category of repetitive events. For example, the statement “playing football with my friends last Saturday” is an example of specific memory, and “our school trip to France” is an example of an over-general memory (Heron et al., 2012; p. 317). In normal conditions and for having healthy psychological functions, the individuals should be able to recall the majority of their autobiographical memories as specific and not over-general (Raes et al., 2009).

Over-generality of autobiographical memory system and chronic thought suppression are associated cognitive mechanisms that can be triggered by traumatic events as a cognitive avoidant coping style. These two maladaptive functions (referred as cognitive avoidance in this study) work in an interrelated way, and they can disturb essential psychological functions (Moore and Zoellner, 2007; Williams et al., 2007; Graham et al., 2014; Bryant, 2015). One of the affected functions due to cognitive avoidance is social problem solving (Beaman et al., 2007; Leahy et al., 2018).

Social problem solving refers to the ability to create and follow effective solutions to cope with problematic social and interpersonal situations (D'Zurilla and Nezu, 2010). Although this ability can be improved by education and learning, it requires some basic cognitive capacities (Madore and Schacter, 2014). Accordingly, effective social problem solving needs access to specific autobiographical memories because past experiences and learnings are important sources of creative problem solving for upcoming situations (Williams, 1996; Barry et al., 2020). Moreover, social problem solving requires a cognitive openness to negative aspects of problematic situations. In other words, for effective social problem solving, negative thoughts and emotions related to the situations should not be avoided (Nezu et al., 2013). As such, cognitive avoidance (including chronic thought suppression and over-general autobiographical memory) can create barriers for the main capacities of social problem solving by disrupting flexible thinking and memory recall (Meir, 1997; Robichaud et al., 2003; Beaman et al., 2007; Kozak et al., 2008).

**Figure 1:**
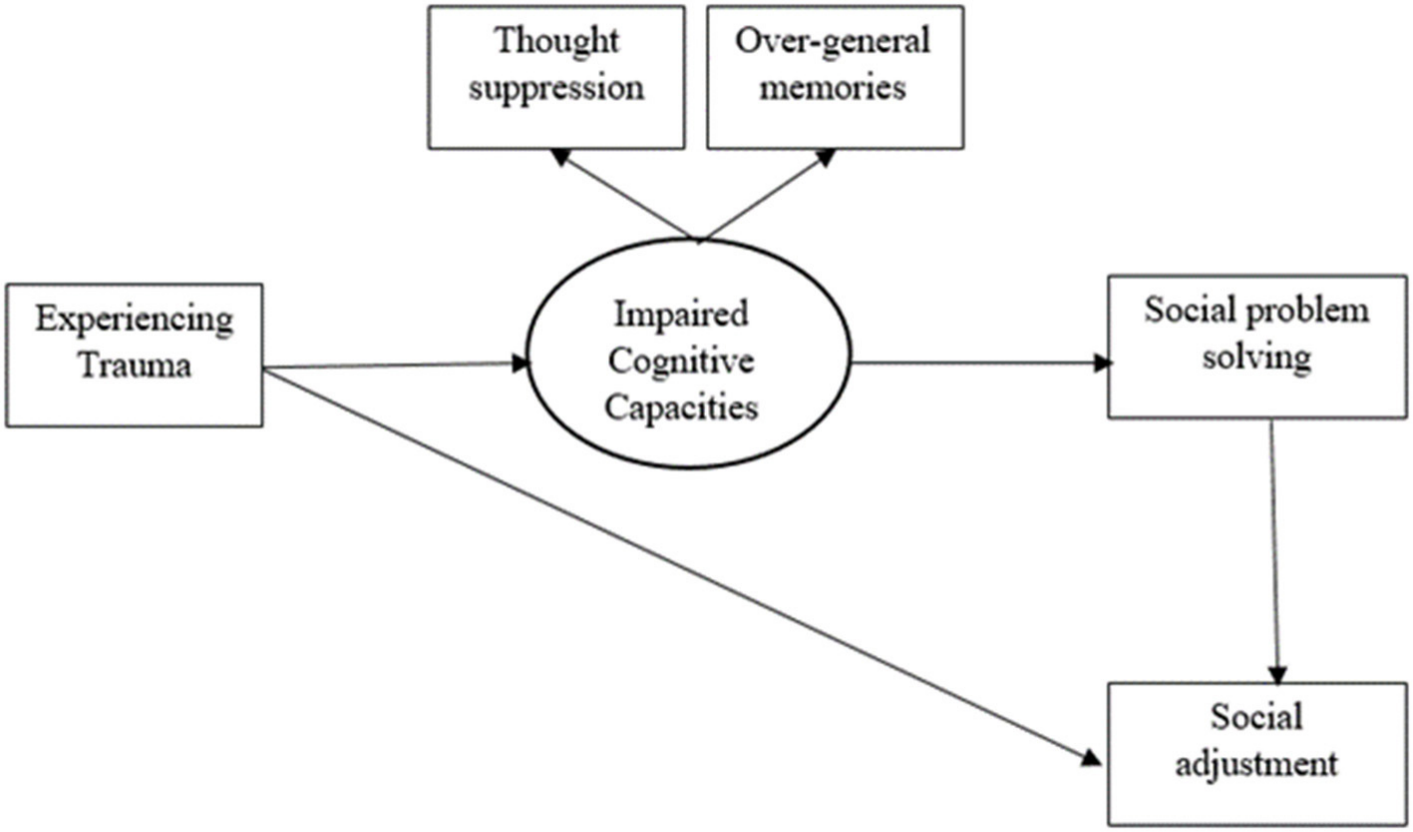
The theoretical model of the relations between trauma and social adjustment. In this model, thought suppression and autobiographical memory have been conceptualized as a unified construct. This is consistent with past theory and research supporting the interrelated functions of these two variables after experiencing trauma (Williams et al., 2007).

In summary, as shown in Figure 1, cognitive avoidance as a coping style in reaction to trauma, can predict lower levels of social problem-solving ability. The final outcome of this process is reduced levels of social adjustment. The reason is that experiencing issues in social and interpersonal situations are inevitable. Thus, if the ability of social problem solving is not effective, the failure to cope with the circumstances can considerably disturb social adjustment (Shure and Spivack, 1980; Asarnow and Callan, 1985; Elias et al., 1986; Fischler and Kendall, 1988; Nezu et al., 2013).

Despite the theoretical background and research evidence supporting different parts of our hypothesized model (Figure 1), no study has investigated this process as a cohesive theoretical framework. However, prior to the current study, the hypothesized model was tested and confirmed in a feasibility pilot study with 96 young individuals (aged 15–29; living in a Middle Eastern country; Ahmadi Forooshani and Izadikhah, 2018).

The hypothesized model can represent important elements in the relation between trauma and social adjustment which can potentially apply to diverse populations of young people from different sociocultural backgrounds. The reason is that this model is focused on the effects of trauma on fundamental cognitive processes that have been considered relevant to basic brain functions (Schauer et al., 2017; Barry et al., 2018). However, there remains debate in the research literature that sociocultural factors such as migration background can contribute to trauma-related difficulties with social adjustment. The rationale for this assertion is that migrant populations are more vulnerable to these difficulties because of potential traumatic experiences before, during and after migration as well as experiencing additional challenges to adjust with a new society (Abebe et al., 2014; Patel et al., 2017; Buchanan et al., 2018; Groen et al., 2018; Hou et al., 2020). However, in past studies there has not been an appropriate comparison between migrant and national young people in the links between trauma and social adjustment. Therefore, it is important to clarify whether the relation between trauma and social adjustment can be applied to diverse populations of young people or if it can be affected by sociocultural factors such as migration background. Therefore, the aim of this study was to identify the links between trauma and social adjustment (Figure 1) in a diverse population of young people. This understanding can inform future research and practice to design specific and effective interventions to promote social adjustment of young people with trauma history.

## Methods

### Participants

The participants were 604 young adults who were living in Australia at the time of data collection. In the sample, 62% reported being born in Australian, while 31% were non-refugee immigrants, and 7% were from refugee backgrounds. All participants were between 18 and 24 years of age with a mean of 19.57 years (*SD* = 1.78). The sample was predominantly female (76.7%) and reported being single (91%). Non-Australian participants were from diverse national backgrounds including 56 countries from all continents (except Antarctica) with no country dominating. The only countries that contributed more than 5% for this group were China (8%), South Africa (7%), Vietnam (7%), India (6%), and the Philippines (6%).

### Procedure

Ethics approval was provided through the Human Research Ethics Committee of the first author's university prior to data collection. Recruitment materials were distributed in print and online forms through universities, social media, social organizations working with young adults, and participants were also recruited through snowball sampling. The eligibility criteria were being aged 18–24 and currently residing in Australia (i.e., not in Australia as a tourist/visitor). The exclusion criterion was not completing the full survey (i.e., more than 5% missing data). Prior to having access to the survey, participants were informed of all the necessary information about their participation, the eligibility criteria, and online, phone, and face-to-face counseling support available as needed. In addition, they had access to the contact details of the first and second authors. The participants could access the questionnaires after indicating their consent to participate. The data were collected through online and print surveys (both in English) from April 2019 to October 2020.

Eight hundred and seventy-nine participants completed the survey. Two hundred and seventy-three participants were excluded as they were not between the ages of 18–24. In addition, two participants were excluded as they had considerable missing data (e.g., a full questionnaire). The final sample consisted of 604 young adults with no missing data higher than 1.3% in the items of the survey. After checking the assumption of Little's test (*p* > 0.05), Expectation Maximation (EM) was used to replace the missing data. This is one of the recommended methods to handle missing data (Schlomer et al., 2010).

### Measures

#### Harvard Trauma Questionnaire (HTQ-5)

This questionnaire was originally developed by Mollica et al. (1992) to assess trauma related symptoms. This is one of the best-known measures for trauma that has been used in hundreds of studies showing good internal consistency (*α* = 0.74–0.94; Darzi, 2017). In this study, we used a 25-item scale of this questionnaire assessing the intensity of trauma. The items could be answered on a Likert scale from 1 (not at all) to 4 (extremely). Higher scores on this questionnaire indicate higher intensity of traumatic symptoms. Good internal consistency was achieved in our study for this questionnaire (*α* = 0.95). An example item is: “recurrent thoughts of memories of the most hurtful or terrifying events.”

#### White Bear Suppression Inventory

Wegner and Zanakos (1994) developed the WBSI to assess chronic thought suppression. The WBSI is a self-report scale consisting of 15 items answered on a Likert scale rated from 1 (strongly disagree) to 5 (strongly agree). Higher scores on this inventory indicate higher levels of chronic thought suppression. Good internal consistency (α = 0.73–0.89) was reported for this inventory in the past studies (Wegner and Zanakos, 1994; Muris et al., 1996). In the current study, high level of internal consistency was computed for this questionnaire (α = 0.93). An example item of the WBSI is: “I wish I could stop thinking of certain things.”

#### Autobiographical Memory Test

In this study, we used the written form of AMT developed by Heron et al. (2012). Respondents are requested to retrieve a personal memory located in a specific time, which is related to the cue word. If the participants recall a memory referring to a long period of time (more than 1 day) or a category of the events, the recorded memory should be coded as over-general. The written form of AMT follows the same scoring system and similar instructions to the original form. Unlike the original form (Williams and Broadbent, 1986), the written form is completely self-report, and it does not need in-person assessment interviews with a tester. Moreover, this form does not include a time limitation to write down the memories. The written form of the AMT includes five positive (excited, happy, lucky, relaxed, and relieved) and five negative cue words (bored, failure, hopeless, lonely, and sad). At the beginning of the test, participants receive a written instruction as well as an example of what they are expected to do. In this study, the frequency of over-general memories was the focus of the assessments, and it could vary between 0 and 10 for each participant. The written form of the AMT has shown acceptable inter-rater reliability (kappa = 0.74–0.93) and its psychometric features have been confirmed through past studies (Heron et al., 2012; Takano et al., 2017). In our study this scoring system showed an appropriate internal consistency (*α* = 0.76).

#### Social Problem Solving Inventory-Revised-Short Form (SPSI-R: S)

This questionnaire was developed by DZurilla et al. (2002) based on an earlier longer version. The 25-item short form evaluates social problem-solving attitudes and skills. Higher scores indicate better social problem-solving ability. The short form of the SPSI-R: S is one of the most relevant instruments to the theoretical approach of social problem solving (D'Zurilla and Nezu, 2010). The psychometric features of SPSI-R: S have been confirmed in the past studies (Hawkins et al., 2009; Li et al., 2016). This questionnaire showed a high level of internal consistency in the current study as well (*α* = 0.86). An example item is: “Before I try to solve a problem, I set a specific goal so that I know exactly what I want to accomplish.” Items are rated on a 5-point Likert scale from 0 (not at all true of me) to 4 (extremely true of me). However, final scores can range between 0 and 20 (i.e., total raw scores are divided by five; DZurilla et al., 2002).

#### Social Adaptation Self-Evaluation Scale

This scale was developed by Bosc et al. (1997) as a short scale for the assessment of social adjustment. The SASS includes 21 items that can be scored from 0 to 3, with higher scores indicating higher levels of social adjustment. Good internal consistency (*α* = 0.74) was reported for this scale in the past studies (Bosc et al., 1997). Similarly, the current study found an appropriate level of internal consistency (*α* = 0.76). An example item is: “How—in general—do you rate your relationships with other people?” which is rated on a 4-point Likert scale from 0 (unsatisfactory) to 3 (very good).

### Data Analysis and Design

In order to investigate the fitness of the hypothesized model (Figure 1) and the direct and indirect relations between the variables, path analysis (based on maximum likelihood estimation method) was used. The analysis was conducted through SPSS-25 and AMOS-25 software. We aimed to do separate path analyses for different populations of young adults (i.e., Australian, non-refugee, and refugee migrant backgrounds). However, the number of refugee participants (*n* = 40) was very small to be considered for a separate path analysis (Schumacker and Lomax, 2010; Wolf et al., 2013). Therefore, we considered refugee and non-refugee immigrants as one group named “migrants,” in our path analyses. Finally, two separate path analyses (i.e., one for Australians and one for migrants) were investigated.

Prior to the main analysis, we conducted a between group analysis to compare the three groups of Australians, non-refugee immigrants and refugees using the means for each variable included in the model (Figure 1). In this preliminary analysis, refugee participants were included as an independent group from immigrants because the number (*n* = 40) was appropriate for a between group analysis (Cohen, 1992). However, since the number of the refugee group was considerably lower than the other two groups, we extracted a random sample of 40 participants from Australian group and another random sample of 40 participants from the non-refugee immigrant group. A simple random selection based on a function of SPSS-25 software was used for this purpose. Then, Multivariate Analysis of Variance (MANOVA) was used to compare the three groups (*n* = 40 per group) in trauma intensity, chronic thought suppression, over-general memories, social problem solving, and social adjustment.

The preliminary between group analysis was necessary to be conducted prior to path analyses because we aimed to consider non-refugee immigrants and refugees as one group (i.e., migrants) for investigating the model. As such, it is important to see the potential differences between these two groups in every single variable. In addition, the potential differences between Australians and non-refugee/refugee migrants in each variable requires clarification to support a better understanding of the results of path analyses.

## Results

### Preliminary Between Group Analysis

MANOVA was used to compare participants from national, non-refugee, and refugee backgrounds (*n* = 40 per group) in all variables of the study while controlling for the effects of gender, age, and marital status. The demographic variables were entered in the model to ensure that the potential differences in the variables of interest are not due to their association with interpersonal differences in gender, age, and marital status. The assumptions of MANOVA were met in terms of equality of covariance matrices (*Box's M* = 31.66; *p* = 0.48) and equality of error variances for trauma (*F* = 0.45; *p* = 0.63), chronic thought suppression (*F* = 2.02; *p* = 0.13), over-general autobiographical memory (*F* = 0.29; *p* = 0.74), social problem solving (*F* = 0.37; *p* = 0.68), and social adjustment (*F* = 0.96; *p* = 0.38).

The results of the overall MANOVA were not significant [Wilks' Lambda *F*_(10, 220)_ = 1.06; *p* = 0.39; *η*^2^ = 0.04] showing that there would not be any significant differences in any of the included variables. Regardless, we checked the follow-up individual ANOVAs as well, but there was no significant result for any variables (*p* > 0.05). In other words, based on the results, the three groups of young people from refugee backgrounds, non-refugee immigrants and Australians were not significantly different in chronic thought suppression, over-general autobiographical memory, social problem solving, and social adjustment. Considering that no comparison was significant and to support a more concise results section, we did not report means and standard deviations for each group. Therefore, descriptive statistics for the variables of interest for the combined group of all participants are presented in Table 1.

**Table 1:** Descriptive statistics for the primary variables in the study for all participants.

**Variables**	***M (SD)***	***Minimum***	***Maximum***
Trauma intensity	50.47 (17.41)	25	100
Thought suppression	52.77 (12.18)	15	75
Over-general autobiographical memory	4.24 (2.10)	0	10
Social problem solving	11.91 (2.55)	2.60	18.20
Social adjustment	43.06 (6.74)	9	63

*n = 604*.

Most of the questionnaires used in this study were not diagnostic instruments. As such, there was not any cut-off point based on which we can categorize the status of participants in each variable. However, the developers of HTQ-5 (used for trauma intensity) suggested that if the total score/25 is ≥ 2.5, that can be associated with a positive diagnosis of PTSD (Berthold et al., 2019). The result of this formula for our data was 2.01, which is below, but close to the cut-off point. In addition, 98% of participants had at least one positive score on the HTQ-5. This demonstrates a considerable frequency and intensity of trauma-related symptoms in our sample from a general population.

Overall, the results of the between group analysis indicate that participants from different groups had similar scores for all the variables of interest. This similarity can imply that similar patterns of relationships between variables may apply to all these groups. However, to ensure a robust and clear investigation, we conducted correlational and path analyses separately for the two groups of Australians and migrants (including immigrants and refugees).

### Path Analyses for the Hypothesized Model

The bivariate correlations between all variables of the model for two groups of participants from national and migrant (including refugee) backgrounds are presented in Table 2. According to the results, in both groups, all the target variables are significantly correlated with each other except for over-general autobiographical memory. This variable had a positive significant correlation with chronic thought suppression in both groups and a significant negative correlation with social adjustment in the migrant group. However, the results of simple correlations do not reject the potential role of over-general autobiographical memory in the hypothesized model because this variable and chronic thought suppression were hypothesized as one latent construct (i.e., cognitive avoidance) in the model. Therefore, if the results of the path analyses confirm these two variables as one latent construct, the direct and indirect relationships between the unified construct with other variables in the model still should be taken to account. The mutual association between chronic thought suppression and over-general autobiographical memory could potentially have significant contributions to the hypothesized mediating effects. Therefore, we tested model fitness without making any changes in the original model (Figure 1).

**Table 2:** Correlational matrix for all the variables included in the hypothesized model.

**Variable**	**Group**	**1**	**2**	**3**	**4**	**5**
1. Trauma	Australian	1				
	Migrant	1				
2. Thought suppression	Australian	0.56^**^	1			
	Migrant	0.48^**^	1			
3. Over-general memory	Australian	0.04	0.23^**^	1		
	Migrant	0.12	0.26^**^	1		
4. Social problem solving	Australian	−0.26^**^	−0.27^**^	0.01	1	
	Migrant	−0.32^**^	−0.21^**^	−0.09	1	
5. Social adjustment	Australian	−0.34^**^	−0.20^**^	−0.01	0.42^**^	1
	Migrant	−0.32^**^	−0.20^**^	−0.13^*^	0.44^**^	1

*The migrant group includes both refugee and non-refugee migrants. n (Australians)= 377. n (migrants) = 227*.

* *p < 0.05*,

** *p < 0.01*.

In terms of model fit, similar results were shown for two path analyses conducted for different groups of the participants. The first path analysis was tested for only Australian participants. The results showed good model fit [*x*^2^ = 0.70 (*p* > 0.05), NFI = 0.99; TLI = 1.02, CFI = 1; RMSEA = 0.00]. The same model was investigated for participants from migrant backgrounds (including voluntary or involuntary migration) and there was also a good model fit [*x*^2^ = 2.15 (*p* > 0.05), NFI = 0.98; TLI = 1.01, CFI = 1; RMSEA = 0.00].

As hypothesized, in both path analyses, cognitive avoidance was confirmed as a latent construct including the two aspects of chronic thought suppression and over-general autobiographical memory. The factor loadings for over-general autobiographical memory were 0.61 in the first and 0.14 in the second path analysis. For chronic thought suppression, the factor loadings were 0.75 in the first, and 0.56 in the second path analysis. Both over-general autobiographical memory and chronic thought suppression had significant contributions to the construct of impaired capacities of social adjustment (*p* < 0.05 for all factor loadings) and to the whole model as well. We found that removing either of these two variables from the model could result in a lack of fitness of the model and a reduction in the intensity and significance of mediating effects in both the path analyses. Therefore, the direct relationships and mediating effects were computed based on the original model as well.

**Figure 2:**
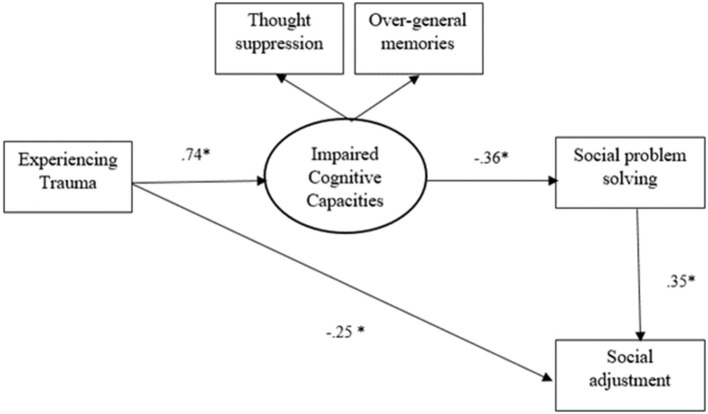
The model of the relationships between trauma and social adjustment in Australian group. ^*^p < 0.001.

**Figure 3:**
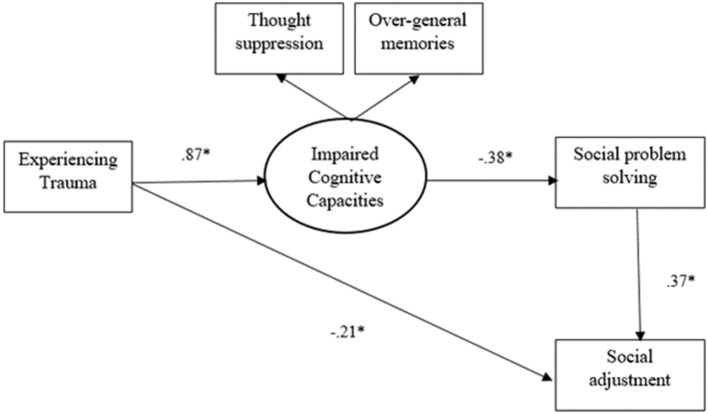
The model of the relationships between trauma and social adjustment in migrant group. The migrant group includes both refugee and non-refugee migrants. ^*^p < 0.001.

As shown in Figures 2 and 3, all direct paths in the hypothesized model were significant in both path analyses. Accordingly, trauma negatively predicted social adjustment and positively predicted cognitive avoidance. The construct of cognitive avoidance negatively predicted social problem solving. And finally, social problem solving positively predicted social adjustment.

Standardized indirect effects with corresponding 95% bias-corrected bootstrapping were computed to investigate mediating effects. All the hypothesized indirect relationships were significant and similar for both path analyses. As indicated in Table 3, cognitive avoidance and social problem solving can significantly mediate the link between trauma and social adjustment. More specifically, the results also confirmed the mediating effect of cognitive avoidance in the relationship between trauma and social problem solving. Moreover, the mediating role of social problem-solving in the relationship between cognitive avoidance and social adjustment was confirmed.

**Table 3:** Standardized indirect effects in the hypothesized model.

**Paths**	**Group**	**Indirect effects**
Trauma → Cognitive avoidance → Social problem solving → Social adjustment	Australian	−0.09^*^
	Migrant	−0.12^*^
Trauma → Cognitive avoidance → Social problem solving	Australian	−0.26^*^
	Migrant	−0.32^*^
Cognitive avoidance → Social problem solving → Social adjustment	Australian	−0.12^*^
	Migrant	−0.14^*^

*The migrant group includes both refugee and non-refugee migrants. n (Australians) = 377. n (migrants) = 227*.

* *p < 0.05*.

## Discussion

The findings of this study confirmed our hypothesized model of the relation between trauma and social adjustment in young adults. Accordingly, if trauma is followed by cognitive avoidance (i.e., chronic thought suppression and over-general autobiographical memory), an impairment in social problem solving is expected, which consequently leads to a significant reduction in the level of social adjustment. To our knowledge, the current study and its related feasibility pilot study (Ahmadi Forooshani and Izadikhah, 2018) are the first research attempts to investigate the hypothesized cognitive model. Our findings were consistent with past theories and research evidence that inspired the proposed model.

In this study, we conceptualized chronic thought suppression and over-general autobiographical memory as a unified construct called cognitive avoidance. This construct explains a maladaptive cognitive coping strategy in reaction to trauma. The conceptualization of thought suppression and over-general memory as a unified cognitive coping strategy, was consistent with the past theory and research (Moore and Zoellner, 2007; Williams et al., 2007). However, the role of these cognitive functions in disturbing the capacities of social adjustment was overlooked in the past literature.

Our results indicated that chronic thought suppression and over-general autobiographical memory as a cognitive avoidance coping style can significantly mediate the link between trauma and social problem solving. This finding was consistent with previous theory and research evidence supporting significant relationships between these variables (Evans et al., 1992; Beaman et al., 2007; Moore and Zoellner, 2007). An explanation for this finding is that effective social problem solving requires a high level of cognitive openness to memories of past experiences and unpleasant aspects of current problematic situations (Robichaud et al., 2003; Beaman et al., 2007). Chronic thought suppression associated with over-general autobiographical memory works against such cognitive openness and therefore hinders social problem-solving ability.

The results of this study support past literature that improving social adjustment requires an improvement in social problem-solving ability (Shure and Spivack, 1980; Asarnow and Callan, 1985; Elias et al., 1986; Fischler and Kendall, 1988; Bell and D'Zurilla, 2009). However, the findings of our study imply that when there is a history of trauma, interventions on social adjustment should not be limited to social problem solving, and they must address the cognitive capacities that might have been affected by trauma. The sequential process suggested in our model, is important to be considered by future studies designing new interventions for social adjustment. Such interventions should target chronic thought suppression and over-generality of autobiographical memory as the first therapeutic targets to build the basic cognitive capacities to promote social adjustment. The improvement of these cognitive functions can facilitate enhancing social problem solving through psychological interventions. Based on our findings, we assert that a comprehensive intervention based on this sequential process is likely to support long-term social adjustment among traumatized young people. Although the pilot findings of this study cannot specify the design of an intervention, they can inform the process of an intervention mapping approach to be followed by future studies.

Finally, the results of this study showed that the relation between trauma and social adjustment can similarly apply to young adults from different backgrounds (e.g., national and migrant). This finding was consistent with past theory and research asserting that the effects of trauma on cognitive processes are based on basic brain functions that are not expected to be significantly moderated by sociocultural factors (Schauer et al., 2017; Barry et al., 2018). Traumatic experiences and their potential effects on social adjustment are not exclusive to immigrant and refugee populations. Based on the results of past studies, 57–75% Australians have at least one traumatic experience and similar rates have been reported in other international studies (Australian Institute of Health Welfare, 2020)6. Our results showed that the vast majority of our participants (from all backgrounds of Australian, immigrant and refugee) reported traumatic symptoms. It seems that the risk of trauma on social adjustment can be predicted for a large proportion of general population of young adults from different backgrounds. This highlights the importance of developing effective interventions to prevent long-term effects of trauma on social adjustment for young people.

### Clinical Implications

We believe that the results of this study can help to fill an important gap in psychological intervention research for social adjustment of traumatized youth. Based on the results of a recent meta-analysis (Ahmadi Forooshani et al., 2019), current standard psychological interventions have limited effectiveness in improving social adjustment of traumatized young people. One of the potential reasons is that the specific relation between trauma and social adjustment and the role of mediating factors have not been sufficiently addressed in the design of interventions (Ahmadi Forooshani et al., 2019). Based on the process investigated in the current study, an effective intervention for social adjustment of young adults with a history of trauma need to specifically target cognitive avoidance (including chronic thought suppression and autobiographical memory), and social problem solving with effective therapeutic measures. We assert that an integrative specialized intervention is needed to rebuild these fundamental capacities in young people with a trauma history. Improving these capacities can help young people to better benefit from related educational resources and mental health services to learn and apply the skills of social adjustment.

### Limitations

We acknowledge that several psychological, social, and cultural factors that have not been included in the current study may contribute to social adjustment. This study was specifically focused on basic cognitive factors that can hinder social adjustment in traumatized youth. The presented model is a pilot theoretical conceptualization that needs to be replicated and extended in future studies to include other relevant emotional, behavioral, and sociocultural factors.

An important limitation for this study was the limited number of people from refugee backgrounds who participated in this study. We kept data collection open for a long period of time (around 1.5 years), but limited numbers of young people from refugee backgrounds participated in this study. The conditions of COVID-19 during the recruitment period contributed to challenges for in person networking with communities and organizations related to refugee populations. This limitation inhibited this study to test the hypothesized model independently for young people with a refugee background. To generalize the findings to diverse populations of young adults, future replications of the current research design should include large equal samples from different populations.

Moreover, a limited proportion of male participants and using self-report questionnaires (without presenting multiple validity and reliability assessments for each instrument) were other limitations of this study. Including a more representative proportion of male participants in future replications can help to investigate potential gender-related differences in the hypothesized variables and model. In addition, using more robust assessment tools such as experimental tests in future studies can reduce the risk of bias in data collection and analyses. Finally, the hypothesized process between trauma and social adjustment needs to be investigated through longitudinal studies. Such studies can provide valuable evidence for potential causal links between trauma and social adjustment.

### Conclusion

Overall, supporting social adjustment for young people has important short- and longer-term implications for their health and wellbeing, and the cognitive model supported herein can provide important information to guide future research and practice. This research presented an evidence-based process explaining how certain cognitive capacities can mediate the effects of trauma on the social adjustment of young adults. This cognitive process can be addressed by future studies to develop effective interventions for social adjustment of young adults with a trauma history.

## Data Availability Statement

In terms of data availability, since our data includes personal autobiographical memories of the participants, it would be against ethical implications to share the data publicly. However, the correspondent author can share the data with researchers requiring the dataset for their studies.

## Ethics Statement

This study was conducted in compliance with the ethical principles of the Australian National Statement on Ethical Conduct in Human Research. Ethics approval was provided through the Human Research Ethics Committee of Queensland University of Technology (Approval Number: 1800001166). The participants provided their written/online informed consent to participate in this study.

## Author Contributions

SA contributed to analyses and writing the original draft. KM, NK, and ZI contributed to review and editing. All authors contributed to conceptualization, investigation, methodology, project administration, and resources.

## Conflict of Interest

The authors declare that the research was conducted in the absence of any commercial or financial relationships that could be construed as a potential conflict of interest.
